# Tumoricidal and Bactericidal Properties of ZnONPs Synthesized Using *Cassia auriculata* Leaf Extract

**DOI:** 10.3390/biom10070982

**Published:** 2020-06-30

**Authors:** Kollur Shiva Prasad, Shashanka K. Prasad, Mohammad Azam Ansari, Mohammad A. Alzohairy, Mohammad N. Alomary, Sami AlYahya, Chandrashekar Srinivasa, Mahadevamurthy Murali, Veena Malligere Ankegowda, Chandan Shivamallu

**Affiliations:** 1 Department of Sciences, Amrita School of Arts and Sciences, Amrita Vishwa Vidyapeetham, Mysuru Campus, Mysuru, Karnataka–570 026, India; 2 Department of Biotechnology and Bioinformatics, School of Life Sciences, JSS Academy of Higher Education and Research, Mysuru, Karnataka–570 015, India; shashankaprasad@jssuni.edu.in; 3 Department of Epidemic Disease Research, Institutes for Research and Medical Consultations (IRMC), Imam Abdulrahman Bin Faisal University, Dammam 31441, Saudi Arabia; 4 Department of Medical Laboratories, College of Applied Medical Sciences, Qassim University, Qassim 51431, Saudi Arabia; dr.alzohairy@gmail.com; 5 National Center for Biotechnology, Life Science and Environmental Research Institute, King Abdulaziz City for Science and Technology, P.O. Box 6086, Riyadh 12354, Saudi Arabia; malomary@kacst.edu.sa; 6 National Center for Biotechnology, King Abdulaziz City for Science and Technology, P.O. Box 6086, Riyadh 12354, Saudi Arabia; salyahya@kacst.edu.sa; 7 Department of Biotechnology, Davangere University, Shivagangotri, Karnataka 577 007, India; chandru.s@davangereuniversity.ac.in; 8 Department of Studies in Botany, University of Mysore, Manasagangotri, Mysuru 570 006, Karnataka, India; botany.murali@gmail.com; 9 Department of Chemistry, Bangalore Institute of Technology, K.R. Road, V V Puram, Karnataka, Bangalore 560 004, India; veenamdy12@gmail.com

**Keywords:** zinc oxide nanoparticles (ZnONPs), *Cassia auriculata*, anticancer property, X-ray photoemission spectroscopy (XRD) pattern

## Abstract

In this work, we aimed to synthesize zinc oxide nanoparticles (ZnONPs) using an aqueous extract of *Cassia auriculata* leaves (CAE) at room temperature without the provision of additional surfactants or capping agents. The formation of as-obtained ZnONPs was analyzed by UV–visible (ultraviolet) absorption and emission spectroscopy, X-ray photoemission spectroscopy (XPS), X-ray diffraction analysis (XRD), energy dispersive X-ray diffraction (EDX), thermogravimetric analysis/differential thermal analysis (TGA-DTA), scanning electron microscopy (SEM), transmission electron microscopy (TEM), high-resolution transmission electron microscopy (HRTEM), and selected area electron diffraction (SAED). The XRD results reflect the wurtzite structure of as-prepared ZnONPs, which produced diffraction patterns showing hexagonal phases. The SEM images indicate that the morphology of as-prepared ZnONPs is composed of hexagonal nanostructures with an average diameter of 20 nm. The HR-TEM result shows that the inter-planar distance between two lattice fringes is 0.260 nm, which coincides with the distance between the adjacent (d-spacing) of the (002) lattice plane of ZnO. The fluorescence emission spectrum of ZnONPs dispersed in ethanol shows an emission maximum at 569 nm, revealing the semiconductor nature of ZnO. As-obtained ZnONPs enhanced the tumoricidal property of CAE in MCF-7 breast cancer cells without significant inhibition of normal human breast cells, MCF-12A. Furthermore, we have studied the antibacterial effects of ZnONPs, which showed direct cell surface contact, resulting in the disturbance of bacterial cell integrity.

## 1. Introduction

In recent years, nanoscience and nanotechnology research has gained ample attention from researchers, as it offers innovative solutions in the fields of materials science, electronics, and medicine [1,2,3]. The significance of nanomaterials is due to their superior physicochemical and biological properties over their bulk phase. Moreover, the size of these materials (<100 nm) offers high surface reactivity due to a higher surface to volume ratio. This distinct property has allowed them to be utilized in applications across many fields, ranging from material science to biotechnology [4,5]. Although there are various methods of synthesizing nanomaterials, both by “top-down” and “bottom-up” approaches, eco-friendly technology for the synthesis of nanomaterials has gained interest due to their ease of acquisition and environmental concerns [6,7].

Among the various metallic nanoparticles, ZnONPs have attracted a lot of interest due to their wide range of applications in optoelectronics, magnetic sensors, environmental protection, biology, and the medicinal industry [8,9,10]. The most important and notable property of ZnO is that its surface is chemically rich in -OH groups, allowing it to slowly dissolve in both acidic and strong basic conditions. It is this property that makes ZnONPs attractive in biomedical applications [11]. Additionally, ZnONPs find roles in medicine as anti-angiogenesis, anti-inflammatory, and anticancer agents [12,13]. Various processes exist naturally to aid in nano- and micro-scaled inorganic material(s) synthesis. These materials of minuscule lengths have laid the foundations for the development of an unexplored and novel platform of nanomaterial biosynthesis. Synthesis of such nanomaterials with the use of naturally occurring plant material abides by the basic principles of green chemistry. Such “green synthesis” protocols are not just environmentally friendly but also non-toxic to existing life forms. Very recently, researchers have reported the use of *Cassia auriculata* flower extract in synthesizing zinc oxide nanoparticles [14]. Furthermore, *C. auriculata*, also known as Tanner’s Cassia, has been reported to offer a wide variety of traditional uses [15,16]. In a recent study, Nawaz et al. reported the anti-breast cancer effect of the ethanolic extract of *C. auriculate* on MCF-7 cell lines, with half maximal inhibitory concentration (IC_50_) values of about 84.56 µg/mL, in an in vitro cytotoxicity assay. They further reported that the use of AgNPs obtained via green synthesis enhanced the cytotoxic potential of the extract by reducing the IC_50_ to 17.25 µg/mL [17]. In another study, the ethanolic extracts of *C. auriculata* leaves were found to inhibit only ~47% of MCF-7 and Hep-2 cells at higher concentrations of 400µg/mL and 500µg/mL, respectively [18]. Furthermore, Prasanna et al. also reported the induction of Bax-mediated apoptosis in the cancer cell lines MCF-7 and Hep-2 [19]. Notwithstanding these studies, very little evaluation of the anticancer activities of *C. auriculata* has been conducted.

Meanwhile, zinc oxide nanoparticles (ZnONPs), a versatile drug delivery tool, have recently been reported to possess significant tumoricidal activity via ROS generation or the caspase-8 and p53 pathway [20,21,22]. However, a better understanding of the mechanistic model and the resultant cellular consequences is essential. Although the metal oxide is considered by the US FDA as a “Generally Recognized As Safe” (GRAS) substance [23], such a categorization typically applies to substances larger than a micron. Hence, it may be deemed necessary to evaluate the cytotoxicity of the same compound in both normal and cancer cell lines. In this study, we put our efforts into re-evaluating the cytotoxic efficacy of ZnONPs prepared using *C. auriculata* extract (CAE) in both cancer (MCF-7) and normal cell lines (MCF-12A).

## 2. Materials and Methods

Precursor anhydrous zinc acetate Zn(OAc)_2_ was obtained from S.D. Fine Chemicals Ltd. (RD Chem, Mumbai, India), and ethanol and acetone were purchased from Merck chemical suppliers (Mumbai, India). Deionized water collected from an ELGA RO water purifier (Elga Veolia, Lane End, UK) was used throughout the experiments. The UV–visible spectrum was obtained using a Lambda 750 UV–visible spectrophotometer (Perkin Elmer, Salt lake, OH, USA). The fluorescence studies were performed on a JOBIN YVON Fluoromax-4 spectrometer (Horiba Ltd, Chiyoda-ku, Tokyo, Japan). Powder XRD was recorded on an X-ray diffractometer using Cu Kα (1.5406 Å) radiation (Bruker, Karlsruhe, Germany). The X-ray photoelectron spectroscopy (XPS) was recorded using a MULTILAB 2000 (THERMO SCIENTIFIC, Los Altos, CA, USA). Scanning electron microscopy (SEM) images and X-ray mapping were recorded on the Zeiss Evo microscope (Carl Zeiss, White Plains, NY, USA). Transmission electron microscopy (TEM) images and SAED patterns were recorded on the JEOL 2100F FEGTEM operating at 200 kV after casting a drop of ZnONP dispersion in ethanol over a Cu grid (Jeol, Akishima, Tokyo, Japan).

### 2.1. Plant Material Collection and Extraction

*Cassia auriculata* leaves were collected from Srirangapatnam, Mysuru, Karnataka, India, (Geographical Coordinates: 12.4237° N, 76.6829° E) in October 2019. The plant was unambiguously identified by Dr. Murali Mahadevamurthy. The leaves were thoroughly washed and dried at room temperature for about 15 days before being ground into a fine powder. The dried plant material (32.8 g of the whole mass) was extracted using water as a solvent by a soxhlet apparatus at 60 °C (1:4 *w/v*). The extract thus obtained was filtered and dried in a hot air oven to yield the crude extract of *C. auriculata* leaves.

### 2.2. Preparation of ZnONPs

To an aqueous solution of anhydrous zinc acetate (1 mM, 1.83 g) in 50 mL of deionized water, the aqueous leaf extract of *C. auriculata* was added (0.32 g dissolved in 25 mL of deionized water) and stirred for 4 h. The formation of pale yellow colored solution from the above reaction mixture implies the formation of ZnONPs. The solution was then filtered using Whatman filter paper and washed with ethanol followed by acetone and dried at 100 °C for 10 h.

### 2.3. Antibacterial Activity of ZnONPs

Test bacteria, namely, *Escherichia coli* (MTCC 7410), *Klebsiella pneumonia* (MTCC 7407), *Ralstonia solanacearum* (MTCC), and *Xanthomonas vesicatoria* (MTCC), were maintained at the Division of Biotechnology, JSS Academy of Higher Education and Research, Mysuru, India. National Committee for Clinical Laboratory Standards (NCCLS) were adopted to carry out the bactericidal activity assay of ZnONPs. The test bacteria used for the study were maintained on nutrient agar media slants at 4 °C and sub-cultured in nutrient broth for 24 h prior to testing. These bacteria served as test pathogens for the antibacterial activity assay.

The as-obtained ZnONPs were subjected to tests for bactericidal activity through the agar well diffusion assay and a growth kinetics study. The nutrient agar plates were prepared using aseptic conditions, and the plates were subsequently streaked with 10^6^ colony forming unit (cfu)/mL of each of the test bacteria using the spread plate method to distribute cells evenly. A sterile cork borer was used to make wells measuring 8 mm in diameter, and wells were filled with 100 μL (2 mg/mL) of ZnONPs. Meanwhile, other wells in the same Petri plates were filled with 100 μL of Milli-Q water and ampicillin as the negative and positive controls, respectively, prior to diffusion at room temperature for 2 h. The diffused plates were then kept in an upright position in an incubator at 37 °C for 12–24 h. After the incubation time, the plates were observed in the inhibition zone and measured accordingly. The minimum inhibition concentration assay was carried out following the protocol published by our research group to find the optimum concentration of the ZnONPs [24].

The test bacteria slabs prepared prior to the experiment were used to conduct the growth kinetics study for the bactericidal activity. The test bacteria were grown in the absence and/or presence of prepared ZnONPs. Bacteria were grown in nutrient broth in the incubator shaker at 100–160 rpm (37 °C), and optical density (OD) was measured at 605 nm at an interval of 30 min using a ultraviolet-visible (UV–vis) spectrophotometer [25].

For the growth kinetics study, the same antibiotic was used as a positive control, and the nutrient broth was used as a negative control. Comparative growth curves of growth kinetics of untreated and treated bacterial culture were prepared for comparison purposes. All of the experiments were conducted in triplicate.

### 2.4. Anticancer Activity

#### 2.4.1. Determination of Anticancer Activity of *Cassia auriculata* (CAE) and ZnONPs

The cytotoxic effects of CAE and ZnONPs were determined by 3-(4,5-dimethylthiazol-2-yl)-2,5-diphenyltetrazolium (MTT) assay. Breast cancer MCF-7 cells procured from American Type Culture Collection (ATCC), Manassas, VA, USA, were cultured in Dulbecco’s Modified Eagle Medium (DMEM) (Invitrogen, Arlington, TX, USA) mixed with 10% fetal bovine serum (FBS) (Invitrogen), and penicillin–streptomycin (100 µg/mL) (Invitrogen) in 5% CO_2_ at 37 °C until confluent. Trypsinization of the cells was carried out using 0.05% trypsin-ethylene diamine tetraacetic acid (EDTA) prior to counting using a hemocytometer. Ten thousand cells/well were plated and incubated in 5% CO_2_ at 37 °C until confluence. Treatments were carried out at CAE and ZnONP concentrations of 10, 20, 40, 80, 160, and 320 µg/mL.

#### 2.4.2. Measurement of Cell Inhibition Using MTT Assay

The MTT assay was conducted according to Denizot and Lang (1986) [26]. After 24 h, the treated cells were fixed using MTT reagent (5 mg/mL) in each well, and cells were incubated at 37 °C for 1 h and centrifuged at 3000 rpm for 5 min. Plates were removed from the centrifuge, and excess dye was washed, and 100 μL of DMSO was added to solubilize the crystal. Optical density (OD) was taken at 570 nm, and the percentage of inhibition was calculated using the formula below.

(1) $$\%\;inhibition=100-\left[\frac{OD\;of\;sample-OD\;of\;blank}{OD\;ofcontrol-OD\;of\;blank\;}\right]\times 100$$

The observations were represented graphically using the Prism 8 statistical analysis tool (GraphPad Software, San Diego, CA, USA).

## 3. Results and Discussion

### 3.1. Absorption and Emission Spectral Studies

**Figure 1:**
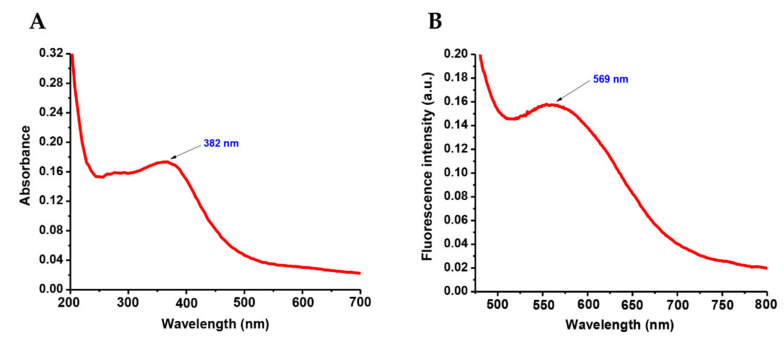
(**A**) The absorption and (**B**) emission spectra of as-obtained ZnONPs.

The UV–visible absorption spectrum of as-obtained ZnONPs was recorded in ethanol (dispersed) and is shown in Figure 1a. The absorption maximum observed at 382 nm was due to the excitation of valence electrons of ZnO arranged in the nanoparticles, typically referred to as the plasmon resonance phenomenon. The emission maximum at 569 nm, as displayed in Figure 1b, was due to band-to-acceptor transitions because of the large binding energy associated with ZnO.

### 3.2. X-Ray Diffraction Analysis

**Figure 2:**
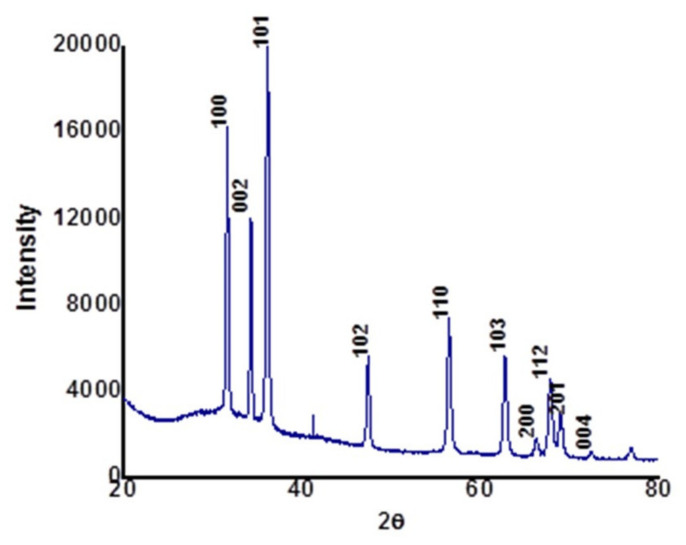
XRD diffraction pattern of as-grown ZnONPs obtained using aqueous extract of *C. auriculata* leaves.

**Figure 3:**
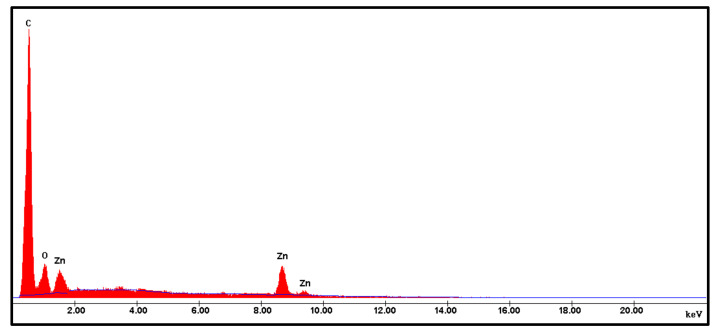
Energy-dispersive X-ray diffraction spectrum of ZnONPs under study.

Figure 2 shows the XRD patterns of calcined ZnONPs synthesized using the aqueous extract of *Cassia auriculata* leaves. The diffraction pattern agrees with the face-centered cubic crystal structure of the hexagonal phase, i.e., wurtzite structure (JCPDS 36-1451). The XRD pattern reveals the crystalline nature of as-obtained ZnONPs. The significant diffraction peaks observed at angles (2θ) 31.98°, 34.53°, 36.28°, 47.68°, 56.54°, 62.94°, 66.52°, 67.94°, 69.12°, and 72.94° correspond to reflection from (100), (002), (101), (102), (110), (103), (200), (112), (201), and (004) planes, respectively. In addition, a less intense peak observed at 2θ = 42.3° reflects the amorphous phase of the biological functional groups from the organic components of the *Cassia auriculata* leaf extract [27]. No impurity diffraction peaks are found in the XRD spectrum, confirming the highest purity of as-obtained ZnONPs [18]. The energy-dispersive X-ray diffraction spectrum of as-synthesized ZnO nanostructures is shown in Figure 3. In the EDX technique, the measurement of a wide energy range (>20 KeV) allows the detection of all elements (with the exception of H and He) at all locations sampled by the beam, which provides a significant advantage with complex microstructures. The peaks related to the presence of Zn and O can be clearly seen in the EDX spectrum, and the percentage chemical composition of the as-obtained material is depicted in Table 1. This was also evident from FT-IR studies (Supplementary Figure S1). The chemical stoichiometry of ZnONPs reported here is affirmed to be Zn:O ≈ 1:1.

### 3.3. Scanning Electron Microscopy (SEM) Analysis

**Figure 4:**
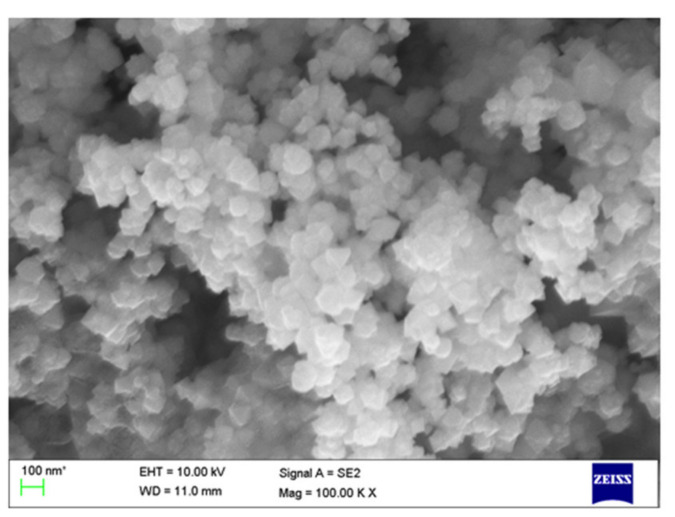
The SEM image of as-obtained ZnONPs using *Cassia auriculata* leaf extract.

The SEM micrograph of as-obtained ZnONPs is displayed in Figure 4. The size and shape of ZnONPs were determined from the SEM image. The SEM results indicate that the as-prepared ZnONPs are composed of hexagonal nanostructures with an average diameter of 20 nm.

### 3.4. X-Ray Photoemission Stroscopy Analysis

**Figure 5:**
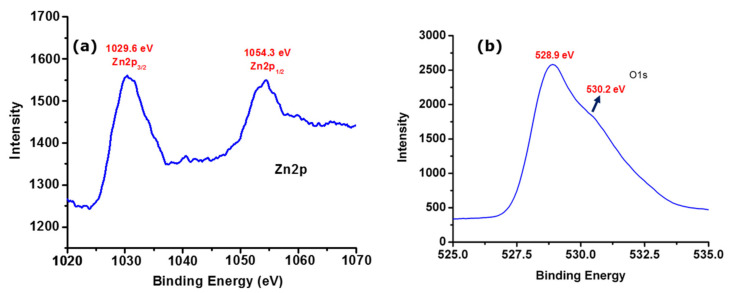
The X-ray photoemission spectra show (**a**) Zn 2p and (**b**) O 1s of as-obtained ZnONPs.

The XPS spectrum of as-obtained ZnONPs was analyzed to investigate the chemical states of Zn. As displayed in Figure 5a, we observed the experimental Zn 2p_3/2_ and Zn 2p_1/2_ photoelectron peaks of ZnONPs prepared at room temperature. The peaks observed at 1029.6 eV and 1054.3 eV correspond to Zn 2p_3/2_ and Zn 2p_1/2_ species, respectively, which coincides with the reported values [28]. Furthermore, spectral deconvolution resulted in an asymmetric peak observed at 528.9 and 230.2 eV in the O 1s spectrum of as-obtained ZnONPs (Figure 5b), which is usually related to the O^2-^ bonding with metals. Thus, in this case, it is a Zn-O crystalline lattice (O_L_) [29,30].

### 3.5. Thermogravimetric Analysis

**Figure 6:**
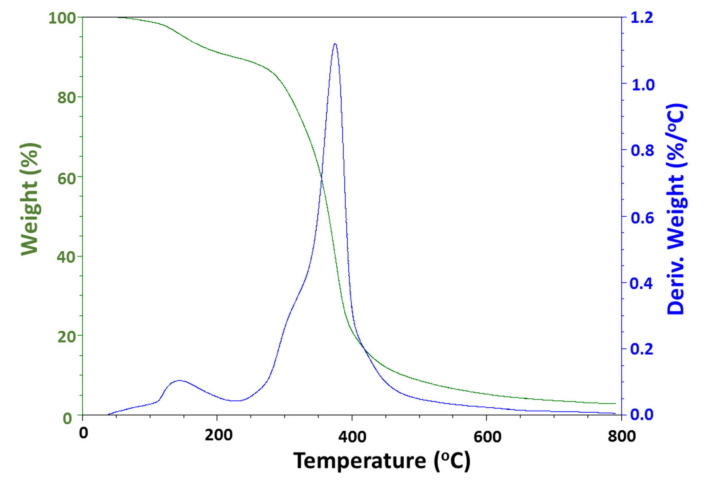
TGA/DTA graph showing the decomposition of as-obtained ZnONPs.

The thermogravimetric analysis and differential thermal analysis of ZnONPs synthesized using the aqueous extract of *C. auriculata* leaves were performed in the temperature range between 25 and 800 °C. The curves of TGA (green curve) and DTA (blue curve) are shown in Figure 6. From the TGA curve, it can be clearly seen that the weight loss starts at ~150 °C, indicating the evaporation of water. A significant loss is observed between 285 and 460 °C, which is due to the decomposition of organic groups present in the sample during green synthesis. Further increase in the temperature leads to no additional decomposition in the sample, which indicates the complete removal of organic substances, leaving behind ZnO. The major exothermic peak observed in the DTA curve between 285 and 460 °C reveals the maximum at 395 °C, which represents the burn-out of organic substance present in the sample. Additionally, there is no significant exothermic or endothermic peak in the DTA pattern.

### 3.6. Transmission Electron Microscopy Investigations

**Figure 7:**
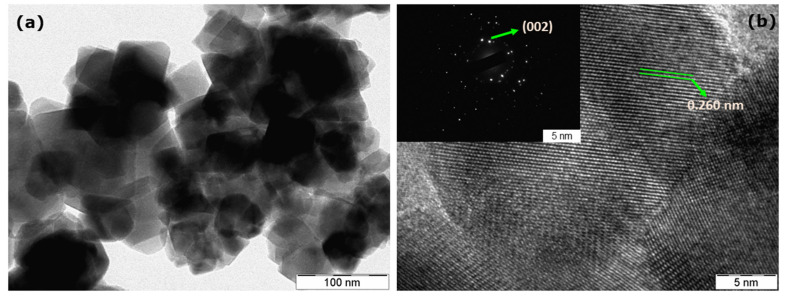
(**a**) TEM and (**b**) HR-TEM images with SAED (inset) of as-obtained ZnONPs.

In order to confirm the size and morphology of as-obtained ZnONPs, TEM analysis was performed. As shown in Figure 7a, the as-obtained ZnONPs have a hexagonal shape with particle sizes between 18 and 20 nm. Furthermore, the crystallinity observed in the XRD spectrum was complemented by high-resolution TEM (HRTEM) studies. As shown in Figure 7b, the observed diffraction lattice fringes in ZnONPs show d-spacing with an inter-planar distance of 0.260 nm between two fringes, which corresponds to d-spacing of the (002) crystal plane of ZnO [20]. Moreover, it has been reported that a decrease in the particle size increases the functionality of antimicrobial and anticancer agents due to the larger surface-to-volume ratio [13].

### 3.7. Bactericidal Activity

**Figure 8:**
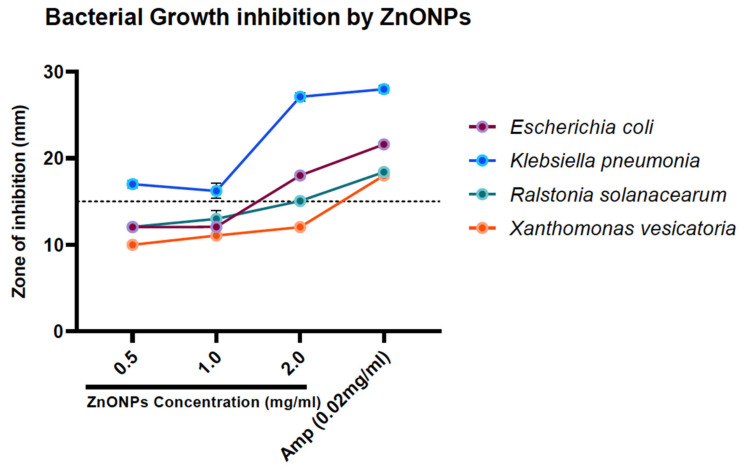
Zone of inhibition graph by ZnONPs against selected bacterial strains.

The disk diffusion assay was performed to analyze the bactericidal activity of the as-obtained ZnONPs against the test bacterial species under study. The results from this study reveal that the antibacterial activity exhibited by the ZnONPs prevented the growth of these bacteria at different concentrations, which can be visualized in the form of a clear zone of inhibition. The bactericidal activity of the ZnONPs was the highest against *Klebsiella pneumonia* in comparison with the standard drug, ampicillin. A moderate zone of inhibition was displayed for *E. coli.* Significant activity was observed by comparing the standard against the plant pathogen selected for the study viz., *Ralstonia solanacearum* and *Xanthomonas vesicatoria* (Table 2 and Figure 8). Furthermore, we compared our results with a literature report on chemically synthesized ZnONPs to account for the significance of as-obtained ZnONPs using an aqueous extract of *C. auriculata* leaves. The results demonstrate that the as-obtained ZnONPs showed a greater antibacterial potency as compared to the commercially available ZnONPs [31].

### 3.8. Study of Growth Kinetics against ZnONPs

**Figure 9:**
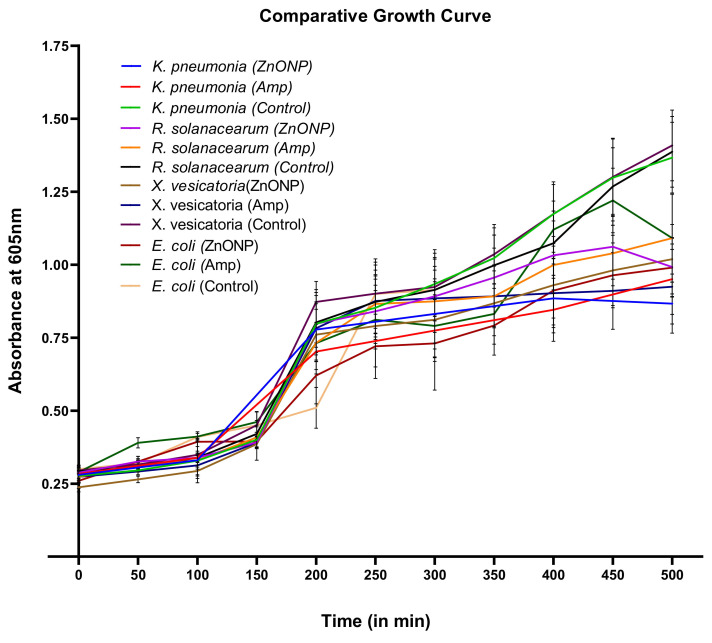
A comparative display of growth curves of test bacteria in the absence and presence of synthesized ZnONPs. Experiments were performed in triplicate.

The growth of all test organisms was analyzed in the presence and absence of ZnONPs, and ampicillin was used as a standard drug against all of the pathogens. A significant decline over time was observed in the growth of all bacterial cultures treated with ZnONPs compared to that of the untreated one. This study suggests that the ZnONPs have activity against the growth of the test organisms. The growth curve (Figure 9) of *Klebsiella pneumonia* displays similar inhibition to that resulting from ampicillin. The bacterial growth of the other test organisms was modestly affected (Supplementary Figure S2).

### 3.9. ZnONPs Sensitized the Cassia Auriculata Leaves Cytotoxicity in MCF-7 Cells

**Figure 10:**
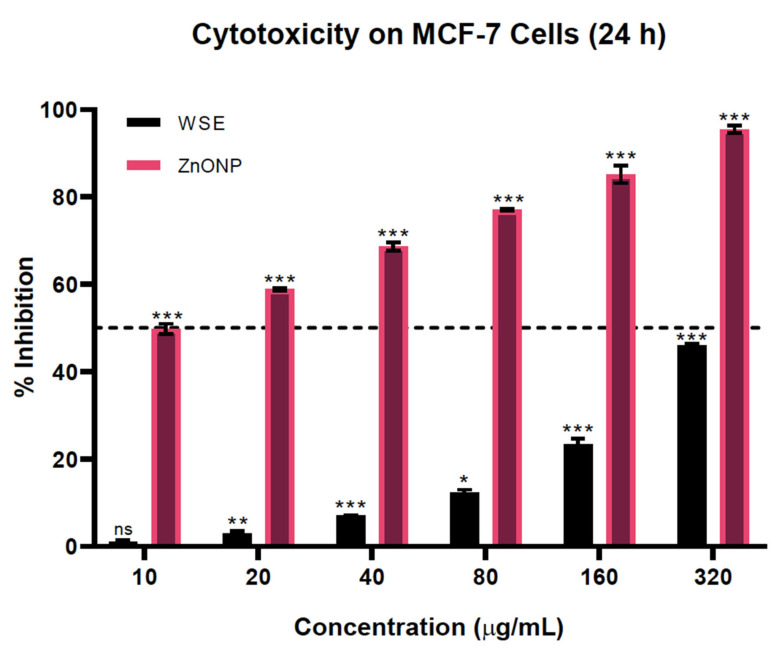
Cytotoxicity of CAE and ZnONPs on breast cancer MCF-7 cells. Results are reported as mean ± SEM for *n* = 3, and a *p*- value of <0.05 was considered to be significant; *p = <0.033, **p = <0.002, ***p = <0.001, ns = not significant.

While all of the treatment groups, involving 10, 20, 40, 80, 160, and 320 µg/mL concentrations of CAE and ZnONPs, showed dose-dependent anti-breast cancer activity, the cytotoxicity of CAE was insignificant at lower concentrations, and nearly 50% viable cells remained even at the very high concentration of 320 µg/mL. However, upon delivery, the ZnONPs caused a drastic shift in the tumoricidal potency of the plant extract. The IC_50_ of the ZnONPs was found to be at least 8-fold lower compared to the independent treatment with CAE. As reported by Prasanna and colleagues, the *C. auriculata* plant extract is indeed cytotoxic to MCF-7 cells only at higher doses [18,19] (Figure 10). However, this can be circumvented with the aid of ZnONPs to enhance the anti-tumorigenic potential of CAE at low doses. Nanoparticle-aided delivery of phyto-compounds has been frequently reported to reduce their IC_50_ value in in vitro as well as in vivo models [25], thereby suggesting that the ZnO nanoparticle-guided delivery of CAE improves its anti-tumorigenic activity.

### 3.10. Neither Cassia Auriculata Leaves nor ZnONPs Showed Significant Toxicity on MCF-12A Cells

**Figure 11:**
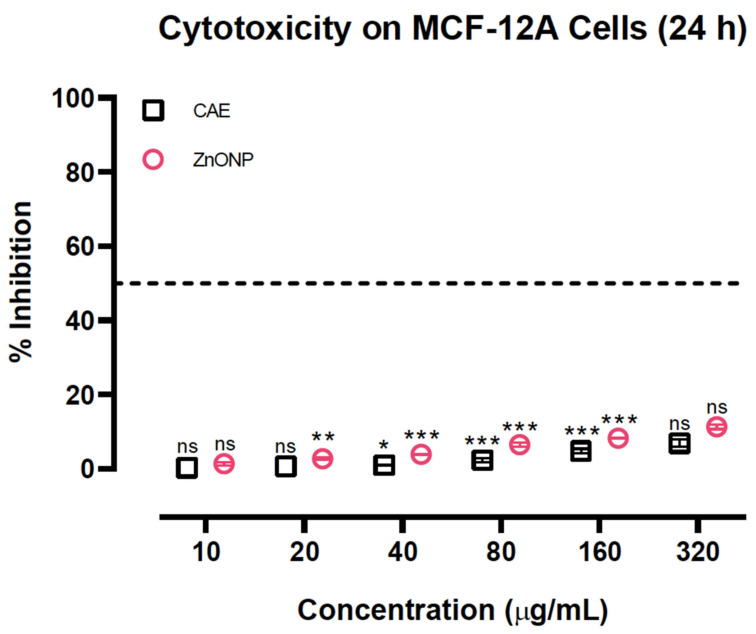
Cytotoxicity of CAE and ZnONPs on normal human breast MCF-12A cells. Results are reported as mean ± SEM for *n* = 3, and a *p-value* of <0.05 was considered to be significant; *p = <0.033, **p = <0.002, ***p = <0.001, ns = not significant.

Although as-obtained ZnONPs enhanced anti-breast cancer activity, no significant growth inhibition was observed in a similar treatment in normal breast cells, MCF-12A. In this first-of-its-kind study on the cytotoxicity of *C. auriculata* in normal human cells, we identified that the plant extract had no noteworthy effect on the growth of MCF-12A cells (Figure 11). Surprisingly, the CAE extract significantly attenuated the normal cell cytotoxicity of ZnONPs, confirming the chemoprotective potential of CAE [26].

## 4. Conclusions

In the present study, we obtained zinc oxide nanoparticles by a convenient green approach using the aqueous extract of *C. auriculata* leaves as reducing and capping agents by continuous stirring for 4 h at room temperature. The ZnONPs synthesized using CAE improved its tumoricidal potential in breast cancer MCF-7 cells while not significantly affecting normal human breast MCF-12A cell growth. Therefore, this implies that the as-obtained ZnONPs are potential therapeutic candidates for breast cancer. However, evaluation of in vivo tumor reduction potential and mechanistic elucidation of the observed anti-tumorigenic effect is deemed necessary for the ZnONPs. Furthermore, the ZnONPs were tested against both plant and animal pathogens, and the potential effectiveness of the nanoparticles resulted in inhibition of all the selected test organisms, as compared with the standard drug. Thus, the presently reported synthetic route and material could find significant importance in pharmaceutical applications.

## Figures and Tables

**Table 1:** The EDX analysis depicting weight % and atomic % of zinc and oxygen elements present in the as-obtained ZnONPs.

Element	Weight %	Atomic %
Zinc	29.32	15.40
Oxygen	19.66	33.63

**Table 2:** The well diffusion assay at different ZnONP concentrations after 24 h incubation at 37 °C. The positive and negative controls were ampicillin (0.02 mg/mL) and Milli-Q water, respectively.

Test Organism	ZnONPs (mg/mL)			Positive Control
	0.5	1.0	2.0	(0.02 mg/mL)
*Escherichia coli*	12.03 ± 0.10	12.06 ± 0.05	18.00 ± 0.30	21.60 ± 0.37
*Klebsiella pneumonia*	17.00 ± 0.40	16.23 ± 0.87	27.10 ± 0.47	28.00 ± 0.45
*Ralstonia solanacearum*	12.06 ± 0.15	13.00 ± 0.98	15.06 ± 0.05	18.40 ± 0.15
*Xanthomonas vesicatoria*	10.00 ± 0.20	11.06 ± 0.10	12.03 ± 0.15	18.00 ± 0.30

## Supplementary Materials

The following are available online at https://www.mdpi.com/2218-273X/10/7/982/s1, Figure S1: FT-IR spectrum of as-obtained ZnONPs using CEA and Figure S2: Comparative display of growth curves of test bacteria in absence and presence of synthesized ZnONPs. Experiments were performed in triplicate.
