# Supplementary material for: Tumoricidal and Bactericidal Properties of ZnONPs Synthesized Using Cassia auriculata Leaf Extract
Source: Biomolecules. 2020 Jun 30;10(7):982. doi: 10.3390/biom10070982 (PMC7407615; doi:10.3390/biom10070982)
Supplement: Supplementary file 1 [file biomolecules-10-00982-s001.pdf]

Supplememtary information

Tumoricidal and Bactericidal Properties of ZnONPs Synthesized Using *Cassia auriculata* Leaf Extract

Kollur Shiva Prasad[1*], Shashanka K Prasad[2], Mohammad Azam Ansari[3*], Mohammad A Alzohairy[4], Mohammad N Alomary[5], Sami AlYahya[6], Chandrashekar Srinivasa[7], Murali Mahadevamurthy[8], Veena Malligere Ankegowda[9], Chandan Shivamallu[2*]

[1]*Department of Sciences, Amrita School of Arts and Sciences, Amrita Vishwa Vidyapeetham, Mysuru Campus, Mysuru, Karnataka – 570 026, India.*

[2]*Department of Biotechnology and Bioinformatics, School of Life Sciences, JSS Academy of Higher Education and Research, Mysuru, Karnataka – 570 015, India.*

[3]*Department of Epidemic Disease Research, Institutes for Research and Medical Consultations (IRMC), Imam Abdulrahman Bin Faisal University, Dammam 31441, Saudi Arabia.*

[4]*Department of Medical Laboratories, College of Applied Medical Sciences, Qassim University, Qassim 51431, Saudi Arabia.*

[5]*National Center for Biotechnology, Life Science and Environmental Research Institute, King Abdulaziz City for Science and Technology, P.O. Box 6086, Riyadh, Saudi Arabia.*

[6]*National Center for Biotechnology, King Abdulaziz City for Science and Technology, Riyadh, P.O. Box 6086, Saudi Arabia.*

[7]*Department of Studies in Biotechnology, Davangere University, Shivagangotri, Karnataka – 577 002, India.*

[8]*Department of Studies in Botany, University of Mysore, Manasagangotri, Mysore 570006, Karnataka, India.*

[9]*Department of Chemistry, Bangalore Institute of Technology, K.R. Road, V V Puram, Karnataka, Bangalore 560 004, India; veenamdy12@gmail.com*

*Correspondence: shivachemist@gmail.com (K.S.P.); maansari@iau.edu.sa (M.A.A.); chandans@jssuni.edu.in (C.S.)

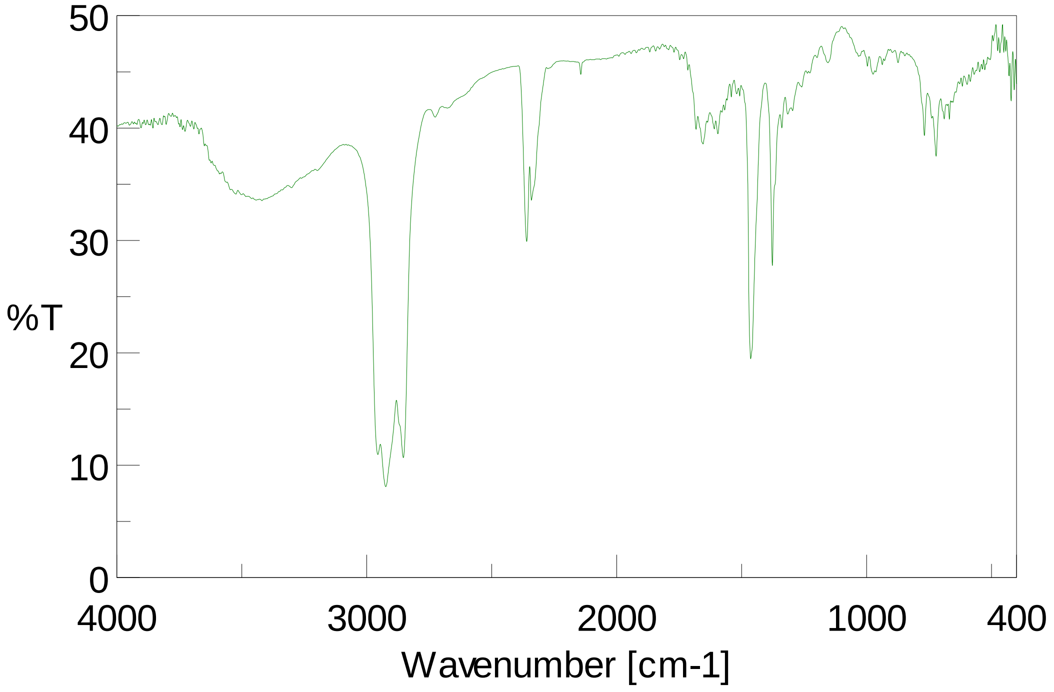

Figure S1: FT-IR spectrum of as-obtained ZnONPs using CEA.

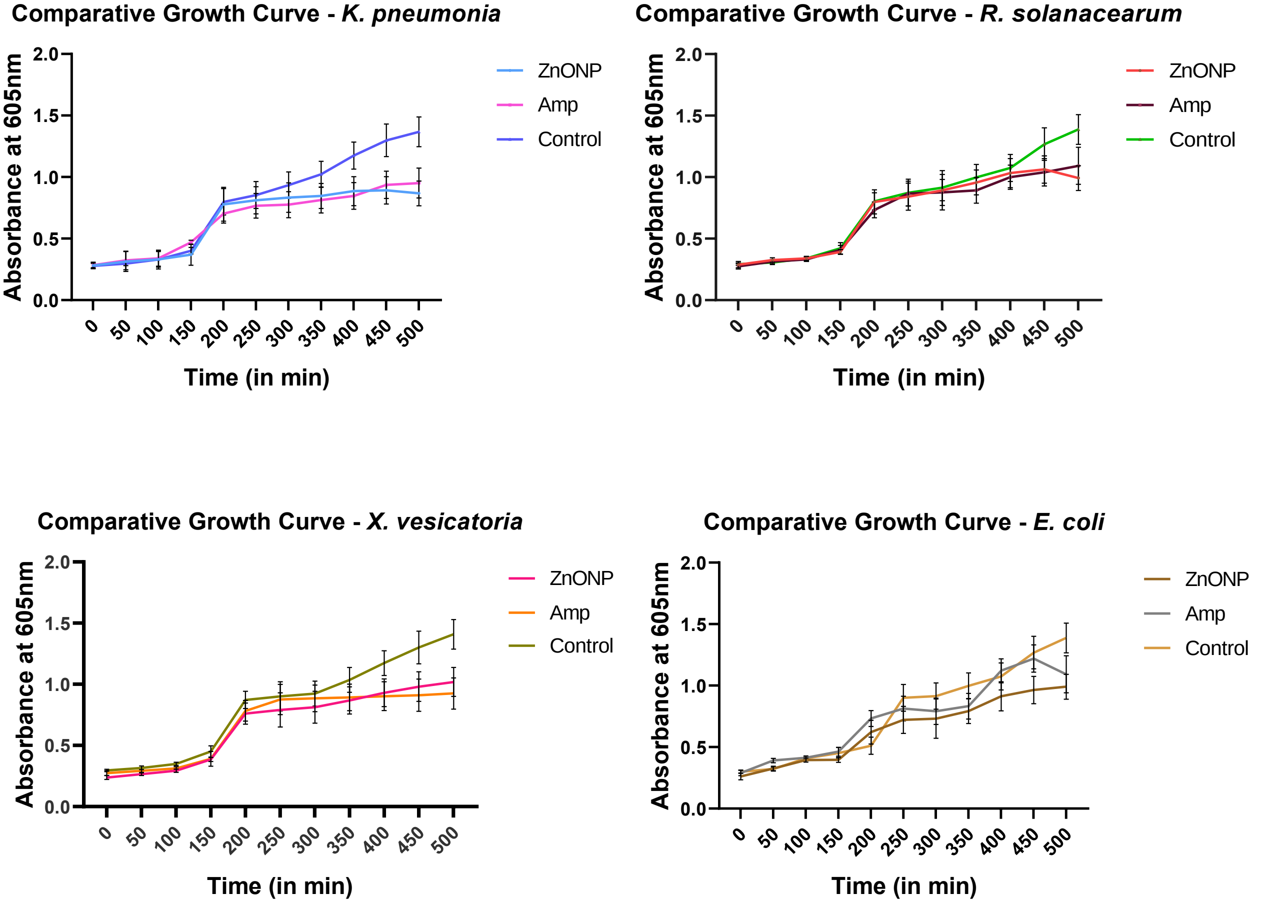

Figure S2: Comparative display of growth curves of test bacteria in absence and presence of synthesized ZnONPs. Experiments were performed in triplicate.
